# Mitochondrial homeostasis: the central hub governing the progression of atherosclerosis

**DOI:** 10.1093/pcmedi/pbag010

**Published:** 2026-03-16

**Authors:** Hao Liu, Shuaiyong Zhao, Huiqin Gao, Yue Wang, Junyan Gao, Ping Guo, Yiting Yang, Wenrui Cui, Shuanglin Zhang, Yaping Shi, Guanxing Xie, Yutong Han, Junya Zhou, Qingqi Zhang, Yunzeng Zou

**Affiliations:** Institute of Advanced Medical Sciences and Huaihe Hospital, Henan University, Kaifeng 475000, China; State Key Laboratory of Respiratory Disease, Guangzhou Municipal and Guangdong Provincial Key Laboratory of Protein Modification and Degradation, School of Basic Medical Sciences, Guangzhou Medical University, Guangzhou 511436, China; Institute of Advanced Medical Sciences and Huaihe Hospital, Henan University, Kaifeng 475000, China; Institute of Advanced Medical Sciences and Huaihe Hospital, Henan University, Kaifeng 475000, China; Institute of Advanced Medical Sciences and Huaihe Hospital, Henan University, Kaifeng 475000, China; Institute of Advanced Medical Sciences and Huaihe Hospital, Henan University, Kaifeng 475000, China; Institute of Advanced Medical Sciences and Huaihe Hospital, Henan University, Kaifeng 475000, China; Institute of Advanced Medical Sciences and Huaihe Hospital, Henan University, Kaifeng 475000, China; Institute of Advanced Medical Sciences and Huaihe Hospital, Henan University, Kaifeng 475000, China; Institute of Advanced Medical Sciences and Huaihe Hospital, Henan University, Kaifeng 475000, China; Institute of Advanced Medical Sciences and Huaihe Hospital, Henan University, Kaifeng 475000, China; Institute of Advanced Medical Sciences and Huaihe Hospital, Henan University, Kaifeng 475000, China; Institute of Advanced Medical Sciences and Huaihe Hospital, Henan University, Kaifeng 475000, China; School of Nursing and Health, Henan University, Kaifeng 475004, China; Institute of Advanced Medical Sciences and Huaihe Hospital, Henan University, Kaifeng 475000, China; Institute of Advanced Medical Sciences and Huaihe Hospital, Henan University, Kaifeng 475000, China; Shanghai Institute of Cardiovascular Diseases and State Key Laboratory of Cardiovascular Diseases, Zhongshan Hospital and Institutes of Biomedical Sciences, Fudan University, Shanghai 200032, China

**Keywords:** atherosclerosis, mitochondrial homeostasis, mitochondrial quality control, mt-DAMPs, immunometabolism, mitochondrial DNA (mtDNA)

## Abstract

Atherosclerosis is a disease centered on chronic inflammation, in which mitochondrial damage plays a key role in its initiation and progression. Traditionally, atherosclerosis is thought to be triggered by cholesterol accumulation, but recent studies have revealed that mitochondrial dysfunction has emerged as an important driving factor by inducing innate immune imbalance. In atherosclerosis, mitochondria undergo changes in membrane permeability, metabolic disorders, and dynamic imbalance due to oxidative stress and other factors, releasing mitochondrial damage-associated molecular patterns (mt-DAMPs). These mt-DAMPs activate innate immune pathways, promote the production of type I interferons and the release of pro-inflammatory factors such as interleukin 1β, and accelerate plaque progression. Mitophagy exerts a protective effect by eliminating damaged mitochondria. Specifically, the PINK1-Parkin pathway labels damaged mitochondria through ubiquitination; mitophagy receptors (such as NIX, FUNDC1, and BNIP3) directly bind to LC3 to initiate ubiquitination-independent mitophagy; and mitochondrial-derived vesicles selectively encapsulate damaged components and target them to lysosomes for degradation. All these processes can reduce mt-DAMP-induced damage and inhibit excessive immune activation. In this review, we summarize that innate immune imbalance caused by mitochondrial damage is a key mechanism for atherosclerosis progression. Mitochondrial quality control clears damaged mitochondria through multiple pathways, alleviates inflammatory responses and plaque burden, and provides potential targets for atherosclerosis treatment. Its precise regulatory mechanisms and drug development are future research directions.

## Introduction

Atherosclerosis is a chronic inflammatory arterial disease. As the primary etiology of cardiovascular diseases and stroke, it has emerged as one of the leading causes of mortality and disability worldwide [1–3]. In terms of pathogenesis, atherosclerosis is characterized by the formation of plaques composed of lipids, connective tissue, and immune cells in the intima of medium and large arteries [2, 4]. When modified low-density lipoprotein (LDL) accumulates excessively in the arterial wall, macrophages phagocytose these lipids and transform into foam cells—a process that not only promotes plaque formation but also exacerbates local inflammatory responses. Beyond the traditional view that metabolic disorders play a dominant role in the development of atherosclerosis, a growing body of evidence in recent years has demonstrated that inflammatory responses and the consequent immune-metabolic imbalance also exert a decisive effect on disease progression [5, 6]. Multiple risk factors, such as lipid overload, oxidized modified LDL, and abnormal shear stress, can induce persistent inflammation in the vascular wall by activating the innate immune response, which promotes foam cell accumulation, fibrous cap thinning, and plaque instability, ultimately triggering severe events including myocardial infarction and stroke [5, 7].

With the rapid developments in the field of immunometabolism, the role of mitochondrial homeostasis regulation in the inflammatory pathology of atherosclerosis has gradually come to light [8, 9]. Mitochondria are not only the hub of energy metabolism but also participate in cellular stress sensing, redox regulation, ion homeostasis maintenance, and the integration of cell death pathways [8]. Thus, mitochondrial damage has been recognized as one of the core drivers of the onset and progression of atherosclerosis [5]. Studies have shown that impaired mitochondrial function in macrophages, vascular endothelial cells (ECs), and vascular smooth muscle cells (VSMCs) commonly leads to increased reactive oxygen species (ROS) production, decreased membrane potential, calcium ion (Ca^2+^) imbalance, metabolic pathway dysregulation, and mitochondrial DNA (mtDNA) release into the cytoplasm [6, 8–10]. These alterations are recognized as damage-associated molecular patterns (DAMPs) by various pattern recognition receptors (PRRs), thereby amplifying the innate immune response [11, 12].

Among these, innate immune pathways such as Toll-like receptors (TLRs), cyclic GMP–AMP synthase–stimulator of interferon genes (cGAS–STING), NOD, LRR, and pyrin domain-containing protein 3 (NLRP3), and absent in melanoma 2 (AIM2) have been demonstrated to be systemically activated in the inflammatory microenvironment of atherosclerosis [2, 6, 9, 11]. For instance, cytoplasmic mtDNA can directly activate the cGAS–STING signaling pathway, inducing the expression of type I interferons (IFNs) and interleukin (IL)-6 [9, 11, 12]. Additionally, mtDNA and oxidized lipids can promote the assembly of the NLRP3 inflammasome, driving the maturation and secretion of IL-1β and IL-18, and forming a persistent inflammatory positive feedback loop [5, 6]. TLR4 activation further enhances nuclear factor-κB (NF-κB)-mediated inflammatory responses and disrupts lipid homeostasis [13]. With the accumulation of mitochondrial damage, these innate immune signaling cascades are continuously reactivated, forming the pathological basis of chronic inflammation in atherosclerosis [5, 6].

Meanwhile, mitochondrial metabolic processes, particularly the functional status of the tricarboxylic acid (TCA) cycle, exert a profound regulatory effect on innate immunity [14]. TCA cycle metabolites, such as succinate, itaconate, and citrate, not only meet the demands of energy production and biosynthesis but also act as signaling molecules to modulate immune responses [9]. For example, succinate promotes the expression of pro-inflammatory cytokines by stabilizing HIF-1α, whereas itaconate exhibits significant anti-inflammatory activity by regulating nuclear factor erythroid 2-related factor 2 (Nrf2) or inhibiting succinate dehydrogenase to limit ROS production [15]. Impaired TCA cycle enzyme activity and electron transport chain (ETC) dysfunction force immune cells to undergo metabolic reprogramming, rendering macrophages more prone to acquiring a pro-inflammatory phenotype and thus directly driving the immunopathological processes of atherosclerosis [14].

Furthermore, abnormalities in mitochondrial calcium homeostasis and dynamics (fusion/fission) serve as important structural bases for driving DAMP release and the initiation of inflammatory signaling [16, 17]. Ca^2+^ overload promotes the persistent opening of the mitochondrial permeability transition pore (mPTP) and changes in membrane permeability, increasing the release of mtDNA and cytochrome c [16]. Excessive fission mediated by dynamin-related protein 1 (DRP1) or impaired fusion caused by mitofusin (MFN)/optic atrophy 1 (OPA1) dysfunction leads to mitochondrial fragmentation, ROS accumulation, and the failure of mitochondrial quality control, further promoting the activation of NLRP3 and cGAS–STING [8, 17]. These lines of evidence reveal that the disruption of mitochondrial homeostasis constitutes a pathological axis of “metabolic disorder–mitochondrial damage–innate immune activation–inflammatory escalation”, which is a key driver of the progressive deterioration of atherosclerosis [5].

## Mitochondrial metabolism in the regulation of atherosclerosis

### Hallmarks of global reprogramming of the TCA cycle in atherosclerosis

**Figure 1:**
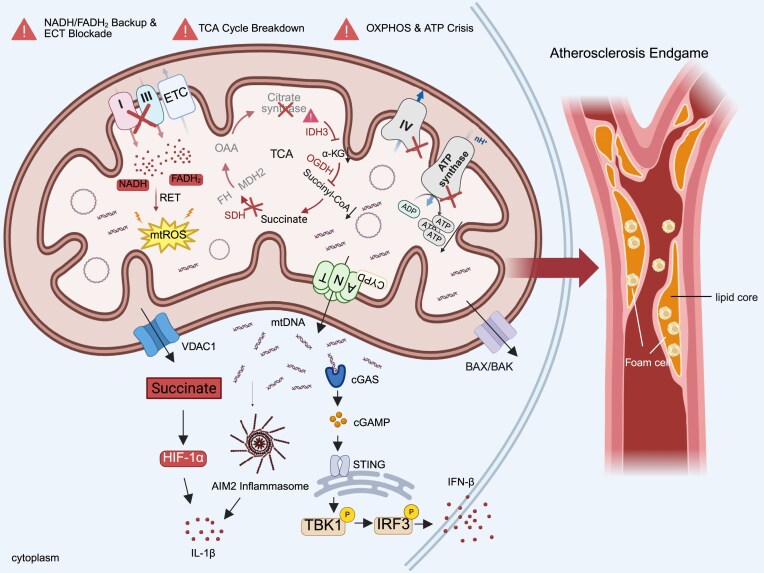
Mechanism by which mitochondrial metabolic dysfunction drives innate immune activation and promotes atherosclerotic progression. This figure illustrates how mitochondrial metabolic dysfunction contributes to the development of atherosclerosis. It first depicts impaired function of the ETC, including the inhibition of Complexes I, III, and IV, leading to the accumulation of reduced nicotinamide adenine dinucleotide (NADH) and reduced flavin adenine dinucleotide (FADH₂) in the mitochondrial matrix, as well as the induction of reverse electron transport (RET) and mitochondrial ROS production. Concurrently, dysfunction of multiple key enzymes in the TCA cycle [e.g. citrate synthase, isocitrate dehydrogenase 3 (IDH3), α-ketoglutarate dehydrogenase (α-KGDH), fumarate hydratase (FH), succinate dehydrogenase (SDH), malate dehydrogenase 2 (MDH2)] results in the accumulation of metabolites such as succinate. On the inner mitochondrial membrane, impaired oxidative phosphorylation (OXPHOS) and reduced activity of adenosine triphosphate synthase lead to insufficient ATP production. Increased OMM permeability mediated by the outer membrane channel protein voltage-dependent anion channel 1 (VDAC1) and BAX/BAK proteins facilitates the release of mtDNA into the cytoplasm. The released mtDNA is recognized by cGAS, which generates cyclic GMP-AMP (cGAMP) to activate STING. Activated STING recruits and phosphorylates TANK-binding kinase 1 (TBK1) and interferon regulatory factor 3 (IRF3), ultimately inducing the production of type I interferon-β (IFN-β). Meanwhile, accumulated succinate stabilizes hypoxia-inducible factor 1 alpha (HIF-1α) and promotes the activation of the absent in melanoma 2 (AIM2) inflammasome, leading to the secretion of Interleukin-1β (IL-1β). The right side of the figure shows how the aforementioned mitochondrial metabolic disorders, activation of the mtDNA–cGAS–STING pathway, and release of inflammatory factors collectively promote foam cell formation in the arterial wall and accumulation of the lipid core, driving the progression of atherosclerotic lesions. The image was generated with full licensed BioRender.com.

As a central hub governing cellular energy metabolism and biosynthesis, the mitochondrial TCA cycle plays a critical regulatory role in the progression of atherosclerosis. Key cellular components within atherosclerotic plaques, including ECs, VSMCs, and macrophages, undergo profound mitochondrial metabolic reprogramming characterized by dysregulated TCA cycle flux and aberrant accumulation of TCA intermediates such as succinate, itaconate, and oxaloacetate. These metabolic disturbances subsequently activate the NLRP3 inflammasome, promote the release of pro-inflammatory cytokines including IL-1β, and thereby drive inflammatory responses and atherosclerotic plaque progression (Fig. 1) [18].

### Role of TCA cycle metabolism in atherosclerosis

Key enzymes and metabolites in the TCA cycle play multiple regulatory roles in the occurrence and development of atherosclerosis, mainly influencing disease progression by regulating immune cell function, inflammatory responses, and metabolic reprogramming. Among them, itaconate is an important immunometabolite generated by the decarboxylation of the TCA cycle intermediate cis-aconitate, catalyzed by aconitate decarboxylase 1 (ACOD1).

In mouse models of atherosclerosis, the expression of itaconate and its synthase ACOD1 is upregulated during atherosclerosis progression; myeloid cell-specific knockout of Acod1 exacerbates inflammatory responses and atherosclerosis lesions, whereas supplementation with itaconate improves plaque stability, characterized by reduced necrotic core size and increased monocyte recruitment [19, 20]. Itaconate exerts anti-inflammatory effects by inhibiting the production of pro-inflammatory cytokines, serving as a key node linking the TCA cycle to the regulation of vascular inflammation [21].

In addition, intermediate products of the TCA cycle exert signal-regulatory functions in atherosclerosis-related immune cells: succinate, as a pro-inflammatory metabolite, can activate the hypoxia-inducible factor 1 alpha (HIF-1α)–IL-1β axis to promote macrophage inflammatory responses; fumarate is involved in regulating the production of cytokines such as IL-10 and type I IFNs in macrophages, thereby affecting the plaque inflammatory microenvironment; and citrate is converted into cytoplasmic acetyl-CoA by ATP-citrate lyase (ACLY), providing raw materials for cholesterol and fatty acid synthesis, promoting hepatic lipid accumulation, and indirectly driving atherosclerosis progression. These metabolites affect plaque stability and disease progression by regulating the metabolic reprogramming and functional status of immune cells such as macrophages and T cells [14, 22].

Furthermore, metabolic reprogramming is associated with plaque phenotypes. Significant perturbations of metabolic pathways exist in atherosclerosis plaques, including abnormalities in the TCA cycle, glycolysis, and amino acid metabolism, which are closely related to high-risk plaque phenotypes such as enhanced inflammation and extracellular matrix degradation. Metabolomic studies have shown that atherosclerosis-associated metabolites are involved in lipid, carbohydrate, and branched-chain and aromatic amino acid metabolism, and are significantly associated with oxidative stress and inflammatory pathways [23]. Targeting the metabolic–immune crosstalk network, such as the itaconate pathway, provides a new strategy for the prevention and treatment of atherosclerosis [22].

In summary, the TCA cycle not only provides cells with energy and biosynthetic precursors, but its key enzymes (e.g. ACOD1, ACLY) and metabolites (e.g. itaconate, succinate, fumarate, citrate) also play core roles in the occurrence, development, and plaque stability of atherosclerosis by regulating immune cell function, inflammatory responses, and lipid metabolism, highlighting the important value of research on atherosclerosis immunometabolism (Fig. 1).

### Mechanism of cell-specific metabolic reprogramming of the TCA cycle in atherosclerosis​

The occurrence of atherosclerosis is directly associated with abnormal TCA cycle metabolism in macrophages, ECs, and VSMCs. In atherosclerosis plaques, activated macrophages exhibit characteristics of TCA cycle remodeling. Inflammatory stimuli such as lipopolysaccharide/IFN-γ induce a two-stage remodeling of the TCA cycle, with transient accumulation of intermediate metabolites (e.g. succinate) in the early stage [24]. Succinate activates the IL-1β signaling axis by stabilizing HIF-1α, significantly enhancing the M1 pro-inflammatory phenotype [25]. Meanwhile, OPA1 deficiency leads to abnormal accumulation of TCA cycle metabolites, impairs NF-κB signal transduction, and hinders the M1 polarization process [26].

Notably, itaconate exerts anti-inflammatory effects, and its level is negatively correlated with the degree of clinical occlusion of human arterial plaques; exogenous supplementation of 4-octyl itaconate can effectively alleviate inflammatory responses. In addition, mechanical stress can disrupt the “arginine metabolism–TCA cycle–mitochondrial function” cascade, further impairing macrophage efferocytosis and exacerbating sterile inflammation [27].

In contrast, TCA cycle dysfunction in ECs directly leads to impaired mitochondrial oxidative phosphorylation (OXPHOS) and increased oxidative stress. Cholesterol loading inhibits TMEM16A expression by upregulating DNA methyltransferase 1 (DNMT1), disrupting the function of calcium-activated chloride channels and impairing vascular barrier integrity. Aberrant activation of TANK-binding kinase 1 (TBK1) can induce endothelial–mesenchymal transition (EndMT) and promote plaque formation; whereas endothelial-specific TBK1 knockdown can significantly inhibit EndMT and attenuate atherosclerosis progression. Furthermore, downregulated Leucine-rich repeat-containing 8A (LRRC8A) expression exacerbates EC cycle disorders, senescence, and oxidative stress, accelerating the vascular aging process. Abnormal lactate metabolism also affects epigenetic regulation through H3K9 lactylation modification, leading to disorders of the “metabolism–epigenetics–transcription” axis and further impairing endothelial function.

VSMCs undergo significant metabolic reprogramming in atherosclerosis. Sirtuin 6 (SIRT6) deficiency promotes VSMC senescence through lipid-mediated oxidative DNA damage and telomere dysfunction, exacerbating plaque instability [28]. Loss of methyltransferase-like 3 (METTL3)-mediated N⁶-methyladenosine (m⁶A) modification induces the transformation of VSMCs to a pro-inflammatory phenotype, enhancing their migration and proliferation abilities [29, 30]. 25-Hydroxycholesterol accumulates in human coronary artery plaques and accelerates the transformation of VSMCs into foam cells through autocrine/paracrine pathways, thereby promoting plaque instability [31].

Notably, VSMC-derived Delta-like ligand 4 signaling can trigger senescence of adjacent cells, reduce collagen synthesis, and directly impair fibrous cap stability. In addition, C-type lectin domain family 4 member A2 (CLEC4A2) deficiency leads to imbalanced homeostasis of vascular-resident macrophages, indirectly affecting VSMC cholesterol metabolism and inflammatory responses [32]. In summary, the metabolic disorders of these cells synergistically promote the formation and progression of atherosclerotic plaques.

### Regulation of innate immunity by TCA cycle metabolic disorders​

Oxidized LDL (oxLDL) impairs mitochondrial metabolism in macrophages, inhibits cholesterol efflux and efferocytosis, and induces the transformation of macrophages into a glycolysis-dependent pro-inflammatory phenotype, thereby abrogating their anti-atherosclerotic capacity [7]. Metabolic reprogramming not only alters energy supply but also affects epigenetic modifications and transcriptional programs through metabolic intermediates, regulating the expression of pro-inflammatory and anti-inflammatory factors. Macrophages and T cells undergo dynamic metabolic remodeling in the plaque microenvironment, which further amplifies inflammatory responses [18]. Mitochondrial NAD depletion can lead to mitotic dysfunction and induce sterile inflammation.

## Mitochondrial dynamics

As the powerhouses of the cell, mitochondria maintain their dynamic equilibrium through continuous fission and fusion processes. This precisely regulated dynamic system involves a variety of core proteins, cytoskeletal components, and transmembrane signaling pathways [33], and its molecular mechanisms can be elaborated at the following levels.

### Mitochondrial dynamics under physiological conditions​

Under physiological conditions, mitochondria maintain dynamic homeostasis through a continuous and precisely regulated process of fusion and fission. This balance is crucial for preserving mitochondrial network integrity, ensuring bioenergetic supply, maintaining calcium homeostasis, and sustaining cell survival [34]. The steady-state morphology of mitochondria is dependent on the dynamic equilibrium between constitutive fission and fusion reactions, which synergistically regulate the shape, number, distribution, and respiratory function of mitochondria [35]. In normal cells, fission and fusion activities are finely coordinated; the absence of either process results in mitochondrial network abnormalities and functional impairment [36].

Core regulatory proteins exhibit stable expression and functional specialization under physiological conditions: mitochondrial fission is primarily mediated by the cytoplasmic GTPase DRP1. Upon phosphorylation, DRP1 translocates to the outer mitochondrial membrane (OMM), binds to receptor proteins (e.g. Fis1, Mff, MiD49/51), and drives membrane constriction and fission [34, 37]. In contrast, the fusion process is cooperatively achieved by the outer membrane GTPases mitofusin 1/2 (Mfn1/2) and the inner membrane GTPase OPA1: Mfn1/2 mediate OMM fusion, whereas OPA1 regulates inner mitochondrial membrane (IMM) fusion and maintains cristae structure [38–40]. The expression levels and activities of these proteins remain relatively constant in healthy cells, collectively preserving the tubular interconnected structure and functional integrity of the mitochondrial network [35, 41].

**Figure 2:**
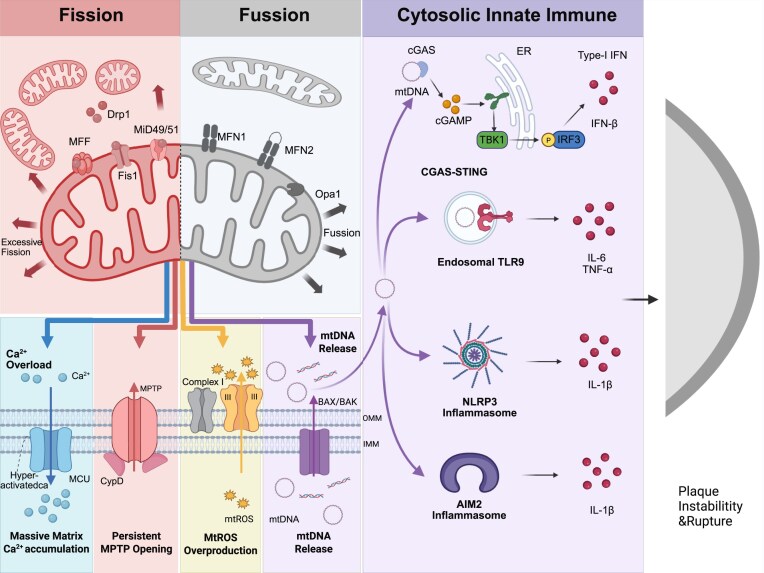
Mitochondrial dynamics imbalance activates cytoplasmic innate immune pathways to promote atherosclerotic plaque instability. This figure illustrates the impact of mitochondrial fission and fusion status on mitochondrial function and cytoplasmic innate immune responses, ultimately leading to atherosclerotic plaque instability and rupture. In the “Fission” section on the left, the mitochondrial fission protein Drp1 is recruited by fission factors including mitochondrial fission factor (MFF) and mitochondrial dynamics proteins of 49 and 51 kDa (MiD49/51), resulting in excessive mitochondrial fission, a process involving the IMM protein fission 1 (Fis1). Excessive fission triggers multiple functional abnormalities, including mitochondrial matrix Ca²⁺ overload via the mitochondrial calcium uniporter (MCU), sustained opening of the mPTP promoted by cyclophilin D (CypD), and excessive production of mitochondrial ROS (mtROS) by mitochondrial respiratory chain Complexes I/III. The “Fusion” section in the center depicts the normal mitochondrial fusion process mediated by MFN1/MFN2 and Opa1. Impaired fusion can induce the formation of B-cell lymphoma-2-associated X protein (BAX)/BCL-2 antagonist/killer (BAK) pores on the OMM, leading to the leakage of mtDNA into the cytoplasm. The “Cytoplasmic Innate Immunity” section on the right illustrates that leaked mtDNA activates multiple inflammatory pathways. Firstly, mtDNA activates the cGAS–STING signaling pathway, inducing the phosphorylation of TANK-binding kinase 1 (TBK1) and interferon regulatory factor 3 (IRF3), thereby promoting the production of type-I IFN and IFN-β. Secondly, mtDNA can be recognized by endosomal TLR9, facilitating the release of IL-6 and tumor necrosis factor-alpha (TNF-α). Additionally, mtDNA and mtROS can activate the NLRP3 inflammasome and AIM2 inflammasome, collectively promoting the production of IL-1β. These inflammatory mediators ultimately lead to the instability and rupture of atherosclerotic plaques, as shown by the structural changes of the vascular pathway in the far right of the figure. The image was generated with full licensed BioRender.com.

Relevant cellular experimental evidence further supports the aforementioned mechanisms. In cultured 143B osteosarcoma cells, confocal imaging and TMRE flow cytometry analysis have confirmed that mitochondrial membrane potential (ΔΨm) is closely associated with dynamic equilibrium under physiological conditions, which is essential for maintaining normal network morphology [42]. In wild-type cells, moderate phosphorylation of Drp1 (e.g. at Ser637) regulates its cytoplasmic–mitochondrial translocation, while stable expression of Mfn2 and OPA1 ensures fusion efficiency [43]. Notably, when key factors of both fission and fusion are simultaneously ablated (e.g. double knockout of Drp1 and Mfn1/2) (Fig. 2), mitochondria restore a tubular network, membrane potential, and respiratory function similar to those of wild-type cells. This finding inversely confirms that fission and fusion must act synergistically to maintain mitochondrial homeostasis under physiological conditions [36]. Additionally, in neuronal and cardiomyocyte models, physiological expression of Mfn2 or OPA1 is indispensable for maintaining mitochondrial cristae structure, OXPHOS efficiency, and cellular stress adaptability [38, 44]. Collectively, these findings demonstrate that mitochondrial dynamics under physiological conditions rely on the precise expression and functional coordination of core regulatory proteins, laying a foundation for understanding the mechanisms underlying its dysregulation in pathological processes such as atherosclerosis.

### Mitochondrial dynamics associated with atherosclerosis

During the pathological progression of atherosclerosis, mitochondrial dynamics imbalance is a key link driving vascular cell dysfunction and plaque progression, in which DRP1, MFN1/2, and OPA1 form the core regulatory axis. DRP1 exhibits a significantly hyperactivated state in atherosclerosis. In ApoE⁻/⁻ mouse models, the expression of DRP1 and the phosphorylation level at Ser616 in vascular ECs and macrophages are increased, promoting its mitochondrial translocation, inducing pathological mitochondrial fragmentation, and exacerbating oxidative stress, inflammatory factor release, and EC activation. Specific inhibition of DRP1 (e.g. using Mdivi-1) can effectively reverse mitochondrial fission, alleviate vascular senescence phenotypes, and inhibit Angiotensin II (Ang II)-induced VSMC phenotypic switching and arterial remodeling, thereby delaying plaque progression [45].

Meanwhile, the mitochondrial fusion proteins MFN1/2 are functionally impaired in atherosclerosis. Various stimuli (e.g. hypoxia, inflammation) lead to downregulated expression or translocation disorders of MFN1/2, impairing the OMM fusion capacity and disrupting mitochondrial network integrity, further damaging energy metabolism, exacerbating cell apoptosis, and weakening the regulation of mitophagy. Studies have shown that restoring MFN2 expression can improve mitochondrial morphology and function, and reduce vascular cell damage [46–48].

As a core factor of inner membrane fusion, OPA1 is significantly downregulated in atherosclerosis. The mRNA level of OPA1 is decreased in human atherosclerosis plaques, and it is closely related to smooth muscle cell function and lipid metabolism pathways. OPA1 deficiency leads to the destruction of mitochondrial cristae structure, decreased membrane potential, and ATP synthesis disorders, and promotes mitochondria-dependent apoptosis. Notably, although specific knockout of OPA1 in hepatocytes can improve systemic lipoprotein metabolism and alleviate atherosclerosis, the downregulated expression of OPA1 in local blood vessels is still positively correlated with plaque instability, suggesting that its protective effect in vascular cells is tissue-specific [49–51].

The synergistic imbalance of the three—excessive fission driven by DRP1 and suppressed fusion function mediated by MFN1/2 and OPA1—collectively contributes to the collapse of mitochondrial quality control, accumulation of ROS, and formation of a chronic inflammatory microenvironment, which has become a key organellar mechanism for the occurrence and development of atherosclerosis (Fig. 2).

### Dynamic changes of mitochondrial dynamics in different stages of atherosclerosis ​

During the pathological progression of atherosclerosis, mitochondrial dynamics imbalance exhibits stage-specific characteristics, mainly manifested by the dynamic evolution of the fission–fusion ratio (Drp1/Mfn2 or Drp1/Opa1). The following is a stage-by-stage elaboration combined with existing research evidence.

The early stage of atherosclerosis (fatty streak stage), is characterized by vascular endothelial injury, lipid deposition, and macrophage infiltration. Mitochondrial dynamics begin to be imbalanced, showing a mild fission dominance. Comparison of coronary artery and aorta samples shows that the abundance of mitochondria-related proteins in early atherosclerosis tissues is significantly lower than that in normal tissues (the difference is particularly obvious in coronary artery samples) [52]. At the initial stage of VSMC phenotypic switching, mitochondrial metabolism and dynamics have already changed, laying the foundation for subsequent plaque formation. In animal experiments, high-fat diet (HFD)-induced mouse models have shown that lipid overload can trigger excessive mitochondrial fission and simultaneously impair lipid droplet–mitochondria contact, suggesting that dynamic disorders exist in the early stage [53].

Entering the middle stage, namely the plaque progression stage, mitochondrial fission is significantly enhanced and fusion is suppressed in macrophages and VSMCs within the plaque, leading to an increased fission–fusion ratio [52]. Studies have confirmed that macrophages in atherosclerosis plaques accumulate abnormal mitochondrial structure and function, accompanied by exacerbated oxidative stress and inflammatory responses [7, 54]. During VSMC phenotypic switching, changes in mitochondrial dynamics (enhanced fission and suppressed fusion) are closely associated with abnormal cellular behavior, promoting the formation of plaque fibrous caps and the expansion of lipid cores [53, 55]. In animal models, inhibition of mitochondrial fission (e.g. using Mdivi-1) can improve lipid droplet–mitochondria interaction, reduce myocardial lipotoxicity, and decrease macrophage infiltration, oxidative stress, and plaque calcification, indirectly suggesting that regulating fission has potential benefits for plaque stability [56]. In addition, increased mitochondrial fission in smooth muscle cells is positively correlated with arterial contraction, while inhibition of fission (e.g. using verapamil) can induce vasodilation and suppress high potassium- or phenylephrine-induced excessive fission, indicating that dynamic imbalance is involved in the regulation of vascular tone [57].

In the advanced stage of atherosclerosis, namely the plaque rupture stage, mitochondrial fragmentation in the plaque is exacerbated, the fission–fusion ratio further increases, the expression of fusion proteins (e.g. Mfn2, Opa1) decreases, and the activity of fission proteins (e.g. Drp1, Fis1) is enhanced. Excessive fission leads to the loss of mitochondrial membrane potential, reduced ATP production, and ROS burst, promoting cell apoptosis and plaque instability [52, 58]. Animal experiments have confirmed that targeted inhibition of macrophage mitochondrial fission can reshape the inflammatory microenvironment and reduce tissue scar formation [59], suggesting that regulating mitochondrial dynamics is expected to stabilize vulnerable plaques.

In summary, the entire course of atherosclerosis is accompanied by the evolution of mitochondrial dynamics from mild imbalance to significant fission dominance, and the increased fission–fusion ratio is positively correlated with disease progression. Existing animal experiments (e.g. HFD mice, Mdivi-1 intervention models, Fis1–Drp1 blocking studies) have provided experimental evidence for targeted mitochondrial dynamics therapy [59]. In the future, it is necessary to further clarify the precise molecular thresholds and cell type-specific changes at each stage to promote the development of precise intervention strategies.

### Cell specificity of mitochondrial dynamics in atherosclerosis

During the progression of atherosclerosis, mitochondrial fission and fusion exhibit distinct cell–specific characteristics in different vascular wall cell types and exert divergent effects on disease progression.

First, in macrophages, dysregulated mitochondrial dynamics directly control inflammatory phenotypes and functional status. Studies have shown that inhibition of mitochondrial fission rebalances the inflammatory microenvironment of macrophages and exerts significant anti–fibrotic effects in mouse scar models, suggesting that mitochondrial dynamics act as a key axis regulating macrophage polarization [59]. In addition, alternative activation of macrophages relies on mitochondrial oxidative metabolism, which is protective for inflammation resolution and anti–atherosclerotic responses [60]. When mitochondrial function is impaired, such as by exposure to oxLDL, macrophages switch from an anti–inflammatory to a pro–inflammatory state and lose critical protective functions including cholesterol efflux and efferocytosis [54].

Second, in ECs, the mitochondrial fusion protein OPA1 is essential for angiogenesis. Under angiogenic stimulation, OPA1 expression is rapidly upregulated to maintain IMM integrity and support the bioenergetic demand of ECs; depletion of OPA1 severely impairs angiogenic capacity [61]. Furthermore, endothelial–specific knockout of mitochondrial calcium uptake regulator MICU1 exacerbates vascular inflammation and accelerates atherosclerosis, whereas MICU1 overexpression is protective, indicating that endothelial mitochondrial calcium homeostasis suppresses atherosclerosis progression by preserving mitochondrial stability [16]. Another study reported that meteorin-like (METRNL) localizes to endothelial mitochondria, and its deficiency aggravates both spontaneous and HFD–induced atherosclerosis, highlighting the central role of endothelial mitochondrial homeostasis in systemic anti–atherosclerotic defense [62]. In addition, aging–associated decline in endothelial mitophagy, such as reduced FUN14 domain containing 1 (FUNDC1) expression with age, is associated with coronary endothelial senescence and increased susceptibility to atherosclerosis [63].

Finally, in VSMCs, alterations in mitochondrial dynamics are closely associated with phenotypic switching. During the transition of VSMCs from a contractile to a synthetic phenotype, mitochondrial metabolism and dynamics undergo profound remodeling [53]. Studies have found that the Fat1 cadherin fragment translocates into VSMC mitochondria and acts as a molecular brake to suppress mitochondrial respiration, thereby modulating VSMC proliferation after vascular injury [64]. Although direct experimental evidence for specific fission/fusion proteins (DRP1, MFN1/2, OPA1) in VSMCs during atherosclerosis remains limited, recent reviews emphasize that rewired mitochondrial metabolism and dynamics are critical drivers of VSMC phenotypic switching [53, 65]. Notably, in other tissues such as the kidney, downregulation of MFN2, but not MFN1, promotes macrophage polarization toward a pro–fibrotic phenotype, implying cell–specific dependency on distinct fusion proteins and providing indirect clues for understanding the functional specificity of mitochondrial fusion proteins in VSMCs.

In summary, macrophages, ECs, and VSMCs exert distinct mechanisms through mitochondrial dynamics to regulate atherosclerosis pathogenesis: macrophages rely on mitochondrial dynamics to maintain anti–inflammatory and reparative functions; ECs preserve mitochondrial homeostasis via OPA1, MICU1, METRNL and other molecules to sustain barrier integrity and anti–atherogenic phenotypes; and VSMCs employ mitochondrial metabolic reprogramming to support phenotypic plasticity. These cell–specific features of mitochondrial dynamics provide a theoretical basis for developing precision therapeutic strategies targeting distinct vascular cell types.

### Mitochondrial dynamics imbalance amplifies inflammatory signaling

DRP1-mediated excessive fission and dysfunctional MFN2 lead to mitochondrial fragmentation, accompanied by ROS accumulation and mt-DAMPs leakage. The bacterial effector protein VgrG4 activates DRP1 via endoplasmic reticulum (ER)–mitochondrion Ca²⁺ transfer, inducing mitochondrial network collapse and NLR family member X1 (NLRX1)-driven ROS production. Fragmented mitochondria are more prone to trigger the NLRP3 inflammasome and cGAS–STING pathway, promoting endothelial activation and plaque instability (Fig. 2) [66]. Mitophagy (e.g. upregulation of Parkin) is compensatorily enhanced in aged vessels, whereas long-term imbalance exacerbates cellular injury [67].

## Regulatory mechanisms and pathological implications of mitochondrial calcium homeostasis

### Uptake mechanism: the core role of the mitochondrial calcium uniporter complex

Ca^2+^ enters the mitochondrial matrix mainly through the mitochondrial calcium uniporter (MCU), which constitutes the core molecule of the uptake mechanism (Fig. 2) [68]. The MCU belongs to a multisubunit complex (MCU complex), including the core subunit MCU and regulatory subunits such as MICU1 and MICU2 [69]. These molecules coordinately regulate the calcium-selective channel: under physiological conditions, when the cytoplasmic calcium concentration rises, the MCU allows rapid Ca²⁺ influx into the matrix, thereby activating mitochondrial dehydrogenases, enhancing the production of NADH and ATP, and supporting cellular energy demands (e.g. muscle contraction or neuronal activity) [68]. Specifically, MICU1 acts as a calcium-sensing “gatekeeper”, inhibiting MCU activity in a low-calcium environment to prevent calcium leakage, and promoting calcium uptake in a high-calcium condition to ensure the efficiency of OXPHOS [70]. This mechanism is particularly important in the myocardium, which can prevent functional disorders caused by calcium imbalance. In addition, the activity of MCU is critical for cellular stress responses: insufficient calcium uptake impairs the adaptive capacity of cells, such as antioxidant defense; while calcium overload may activate the mPTP and trigger cell death pathways, which has been confirmed in neurodegenerative diseases such as Alzheimer’s disease (AD) (involving the interaction between β-amyloid and tau proteins) [71]. The voltage-dependent anion channel 2 (VDAC2) in the OMM also participates in calcium signal transmission. VDAC2 forms mitochondria–ER contact sites through contact with the ER, facilitating the release of ER calcium into mitochondria and affecting the dynamics of calcium uptake [72].

### Extrusion mechanism: regulation of the sodium–calcium exchanger and related pathways

Ca^2+^ extrusion is mainly achieved through the mitochondrial sodium–calcium exchanger (NCLX), which is a key molecule for calcium efflux. In excitable tissues such as the myocardium and brain neurons, NCLX is responsible for exporting Ca²⁺ from the mitochondrial matrix to the cytoplasm to maintain calcium homeostasis and cellular excitation–contraction coupling [73]. The specific pathways include: NCLX drives calcium extrusion by utilizing the transmembrane sodium gradient (Na⁺ concentration difference) to regulate cytoplasmic calcium levels; recent studies have found that the TMEM65 protein may act as a functional analog of NCLX and participate in the calcium efflux mechanism, but its molecular details need to be further confirmed [74, 75]. This process is crucial for ATP production and cellular survival: insufficient calcium extrusion leads to matrix calcium accumulation, triggering oxidative stress and mitochondrial dysfunction (e.g. decreased membrane potential and elevated ROS), and then activating mPTP and caspase-dependent apoptotic pathways [76]. In pathological settings such as AD or myocardial ischemia-reperfusion injury, NCLX dysfunction exacerbates calcium overload, making it a therapeutic target (e.g. restoring homeostasis by regulating calcium pathways via Piezo1) [77]. In addition, the imbalance of calcium extrusion modulates local signals (e.g. calcium-dependent enzyme activity) by affecting the spatially restricted distribution of mitochondrial calcium signal transmission, and plays an important role in cell differentiation, immune responses, and tumor development.

### Molecular and signaling pathways of calcium homeostasis in disease

The dynamic equilibrium of mitochondrial calcium homeostasis involves the crosstalk of multiple molecular regulations and pathways. Key regulatory molecules include the following. MICU1 and MICU2: in addition to being regulatory subunits of the mitochondrial calcium uniporter (mtCU) complex, MICU1 maintains endothelial calcium homeostasis in vascular inflammation and inhibits mitochondrial ROS accumulation associated with calcium imbalance [16]. Loss-of-function mutations in MICU1 cause neuromuscular disorders, impairing neuronal metabolism and calcium signal transmission [78]. Miro1: mutations in the mitochondrial Rho GTPase 1 (Miro1) protein at the calcium-binding domain (e.g. the p.R272Q mutation) disrupt calcium homeostasis, trigger the activation of calcium-dependent kinases, and lead to neurodegeneration (e.g. α-synuclein accumulation and dopaminergic neuron loss) [79]. Signal transducer and activator of transcription 3 (STAT3) and mitochondria-associated membrane structures: STAT3 is localized on mitochondria-associated membrane, indirectly regulating calcium flux and influencing inflammatory and aging processes (e.g. alleviating inflammation-related calcium imbalance by inhibiting the STAT3 pathway) [80]. Related signaling pathways are tightly coupled with mitochondrial function. Energy metabolism pathways: calcium uptake activates TCA cycle enzymes via the MCU, increasing the NADH/NAD⁺ ratio and promoting ATP production; however, imbalance (e.g. calcium overload) reverses this process, resulting in energy deficits, inducing reductive stress in neurons and reducing cellular viability [81]. For example, studies have shown that inhibition of MCU (e.g. with mcui4) can restore calcium homeostasis and energy output [82]. ROS and cell death pathways: disruption of calcium homeostasis increases mitochondrial ROS levels, forming a positive feedback loop by which high calcium activates mPTP, leading to membrane potential collapse and cell apoptosis, which is highlighted in neurodegenerative and tumor contexts (e.g. the linkage between calcium homeostasis dysregulation and mPTP opening in AD) [83]. In addition, calcium signaling interacts with mitochondrial dynamics (e.g. fusion/fission): DRP1 promotes mitochondrial fission and affects calcium distribution; inhibiting DRP1 with Mdivi-1 or overexpressing MFN2 can restore calcium homeostasis and mitochondrial function, protecting cells from oxidative damage (Fig. 2) [84]. Autophagy and quality control pathways: calcium imbalance triggers mitophagy to clear damaged mitochondria, but defective autophagy leads to calcium accumulation and exacerbates disease pathology (e.g. MCU upregulation in hepatotoxicity and neurodegeneration) [85]. Calcium homeostasis also integrates with intracellular calcium buffering systems (e.g. phosphoinositide signaling) to regulate phospholipase C-dependent signals, affecting global cellular calcium dynamics [86].

### Calcium homeostasis imbalance activates immune pathways

Loss of MICU1 in ECs results in mitochondrial calcium overload, exacerbating vascular inflammation and atherosclerosis, whereas MICU1 overexpression is protective [16]. In VSMCs, cholesterol induces MCU–mediated calcium overload and lipid accumulation, and MCU inhibitors alleviate atherosclerosis. Dysregulated calcium signaling promotes the opening of the mPTP, causing the release of mt–DAMPs, and participates in the establishment of trained immunity—monocytes/macrophages acquire a persistent pro–inflammatory phenotype following endogenous stimuli (e.g. modified lipoproteins), which represents a key mechanism underlying chronic inflammation in atherosclerosis (Fig. 2) [87, 88].

## Mitochondrial membrane permeability dysregulation

### Alterations in OMM permeability

Apoptosis holds a unique position in the process of programmed cell death, playing a prominent role in eliminating harmful cells, selecting dominant cells, and maintaining the body’s homeostasis. Mitochondrial apoptosis is the most common form of programmed cell death in humans, a process regulated by B-cell lymphoma-2 (BCL-2) [89]. BCL-2 is an oncogene activated by chromosomal translocation in human follicular lymphoma, and the BCL2 family members BCL2-associated X protein (BAX) and BCL2 homologous antagonist/killer (BAK) interact to jointly regulate mitochondria and govern the mitochondrial intrinsic apoptotic pathway for programmed cell death [90]. Upon exposure to stress signals, BAK and BAX are activated and oligomerize on the OMM, leading to OMM permeabilization (MOMP) and subsequent release of pro-apoptotic factors, primarily cytochrome c [91], in addition to the pro-inflammatory factor mtDNA [92, 93]. Mitochondria possess their own DNA located in the mitochondrial matrix [94]; during OMM permeabilization, a small number of BAX/BAK form pores to trigger cytochrome c release, while most BAX on the OMM is recruited after cytochrome c release to form large BAX/BAK pores. These large BAK/BAX pores allow the IMM to herniate into the cytoplasm, carrying mitochondrial matrix components including the mitochondrial genome, at which point mtDNA is also released from mitochondria to exert its effects in the cytoplasm (Fig. 2) [95, 96].

In addition to BAX/BAK forming large pores to facilitate mtDNA release from mitochondria into the cytoplasm, the VDAC also serves as a pathway for mtDNA release [97]. VDAC plays a key role in the OMM, controlling metabolic processes between mitochondria and the cell, and regulating Ca²⁺ influx, cell death [98], and inflammasome activation [99]. Under oxidative stress stimulation, VDAC can oligomerize to form VDAC oligomers, which in turn form large OMM pores [100]. The difference between the two is that large BAX/BAK pores function under apoptotic or extreme stimulation conditions, whereas VDAC promotes MOMP and mtDNA release upon moderate stress stimulation without cellular apoptosis, and the released mtDNA exists as short, small fragments independent of Ca²⁺ flux [101]. Furthermore, after cleavage by inflammatory caspases, the N-terminal fragment of gasdermin D (GSDMD) can bind to cardiolipin on the OMM and permeabilize both the IMM and OMM, promoting mtDNA release and ROS production [102]. This pathway is independent of the BCL-2 family or mPTP, and represents a key inflammation-dependent mechanism for mtDNA release in the inflammatory microenvironment of atherosclerosis [103]. Of note, GSDMD not only targets mitochondria but also amplifies inflammatory responses and cellular injury by forming pores in the plasma membrane through the canonical pyroptosis pathway. Upon cleavage by inflammatory caspases, the N–terminal domain of gasdermin family proteins (e.g. GSDMD) translocates to the plasma membrane and binds to acidic phospholipids (e.g. phosphatidylethanolamine) to form large β–barrel pores with a diameter of ∼21 nm. This leads to increased membrane permeability, disrupted ionic gradients, and consequently induces pyroptosis and the release of pro–inflammatory cytokines including IL–1β [104–106]. This process not only disrupts cellular homeostasis but may also indirectly impair mitochondrial function, thereby promoting macrophage death and plaque instability in atherosclerosis [107].

### Alterations in IMM permeability

Dysregulation of the permeability of the OMM and the IMM is the primary cause of mtDNA release [108]. The main mechanism by which mtDNA is released across the IMM is the opening of the mPTP, a pore that allows the passage of short mtDNA fragments [109]. The mPTP is a non-specific pore located on the IMM, whose functional state is controlled by numerous regulators and proteins, and various endogenous and exogenous factors have been shown to modulate its opening. On the IMM, the MCU regulates Ca^2+^ concentration [110] between mitochondria and the cell, and Ca^2+^ was the first identified effective modulator of mPTP. Under conditions of mitochondrial calcium overload, the pore opens, allowing solutes of < 1.5 kDa to pass into and out of mitochondria [111, 112]. Pore opening causes dissipation of the mitochondrial membrane potential, organelle swelling, rupture of the mitochondrial cristae structure, and ultimately outer membrane rupture, leading to mitochondrial dysfunction [113] and release of contents including mtDNA.

Regarding research on the molecular structure of the mPTP, the pore is currently believed to consist of three basic components: the VDAC on the OMM, the adenine nucleotide translocase (ANT) on the IMM, and cyclophilin D (CypD), a peptidyl-prolyl isomerase in the mitochondrial matrix. Subsequent studies have shown that several proteins located on the IMM are components of the mPTP and regulate mitochondrial inner membrane permeability, including ANT [114], F1F0 ATP [115] synthase, and SPG7 [116], which affect and regulate ion transport [117] between the cytoplasm and mitochondria via the mPTP, although the mechanisms by which these proteins regulate IMM permeability remain unclear. Recent studies have identified that the prohibitin 1 (PHB1) protein on the IMM regulates the mPTP, thereby inducing mtDNA release. PHB1 is localized on the IMM and interacts with PHB2 to jointly maintain mitochondrial integrity and function [118]. Loss of PHB1 leads to a reduction in mtDNA content in mitochondria [119], promoting inflammation and increasing susceptibility to tissue damage [120]. It also mediates the degradation of mitochondrial proteins by negatively regulating the proteolytic activity of the m-AAA protease complex [121]. The m-AAA protease complex assembles as a heterohexamer with AFG3-like protein 2 (AFG3L2) and SPG7 [122]; further studies have found that SPG7 interacts with CypD, and CypD, as an important component of the mPTP, maintains the functional state of the pore [123]. PHB1, as a core factor, controls mPTP formation and mitochondrial inner membrane permeability by regulating the interaction between SPG7 and AFG3L2; mPTP opening triggers VDAC oligomerization on the outer membrane and pore formation [124], thereby controlling mtDNA release and further inducing downstream inflammatory responses (Fig. 2) [125].

### Effects of mitochondrial matrix membrane permeability

For mtDNA to be released from mitochondria into the cytoplasm, it must cross both the IMM and OMM. In addition to the direct regulation of the two membranes, matrix proteins in the mitochondrial matrix may regulate mtDNA release by modulating the internal mitochondrial environment, affecting the permeability of the IMM and OMM, or participating in intracellular signal transduction pathways [126].

CypD is a peptidyl-prolyl isomerase encoded by the peptidyl prolyl *cis*-*trans* isomerase F (ppif) gene, localized in the mitochondrial matrix, and is also a component of the mPTP [113]. Early studies suggested that CypD interacts with the key protein ANT to initiate mPTP opening. It is now recognized that Ca²⁺ is the most potent agonist triggering mPTP opening [127]. A key step in the action of Ca²⁺ is inducing a conformational change in ANT, and the binding of CypD to ANT promotes mPTP opening [128]. Further research on CypD has found that CypD can be acetylated at specific sites, and acetylation inhibits the deacetylase SIRT3, thereby promoting the binding of CypD to ANT. Under normal physiological conditions, CypD is localized in the mitochondrial matrix, but under oxidative stress or ischemia-reperfusion, CypD translocates to the IMM and undergoes a conformational change, thereby affecting mPTP formation, promoting mPTP opening, and accelerating the cell death process [129].

Under oxidant or stress-induced damage, CypD can recruit misfolded proteins; when the aggregation of recruited proteins exceeds the threshold of CypD, it induces mPTP opening [130]. Under physiological conditions, the mPTP is closed, the IMM is only selectively permeable to certain metabolic substrates and ions, and the mitochondrial transmembrane potential is intact. As mentioned earlier, pore opening causes organelle swelling, outer membrane rupture, mitochondrial dysfunction, release of mitochondrial contents, and release of mtRNA into the cytoplasm.

### Regulation of mitochondrial permeability by cell membrane receptors

The cell membrane is equipped with specific receptors known as PRRs, which constitute a crucial component of the body’s innate immune system. These PRRs can sensitively detect the presence of viral molecules, and upon detection, they rapidly trigger the body’s immune response, mounting a timely defense in the early stages of pathogen infection. This response initiates signaling pathways for the body’s inflammatory response and antiviral immunity, thereby effectively protecting the host from infection [131].

As key components of the immune system, PRRs include TLRs, retinoic acid-inducible gene I (RIG-I)-like receptors, NOD-like receptors (NLRs), and intracellular DNA and RNA sensors [132]. Among them, TLRs are germline-encoded type I transmembrane glycoprotein PRRs; the TLR family is one of the most well-characterized PRR families, responsible for sensing invading pathogens in the extracellular space as well as in intracellular endosomes and lysosomes. TLRs can recognize different pathogen-associated molecular patterns, and by controlling multiple functions of antigen-presenting cells, lead to the activation of innate immune responses and subsequent initiation of adaptive immune responses [133]. However, while exerting immune functions, TLR4 may promote ischemia/reperfusion-induced neuronal apoptosis by inhibiting the Phosphatidylinositol 3-kinase/protein kinase B-glycogen synthase kinase 3β (PI3K/AKT-GSK-3β) signaling pathway or activating caspase-3 [134].

Meanwhile, after PRR activation, lipopolysaccharide can affect the expression levels of Bax and Bcl-2 proteins by activating the TLR4-PI3K/AKT-GSK-3β signaling pathway, altering mitochondrial membrane permeability, thereby triggering the active caspase-3 and mitochondrial apoptotic pathways, and ultimately promoting neuronal apoptosis [135]. As mentioned earlier, during apoptosis, mtDNA is released from mitochondria into the cytoplasm through the large pores formed by BAX/BAK (Fig. 2) [136]. Therefore, in addition to using mtDNA as a DAMP to exert immune functions, PRRs may further promote mtDNA release; moreover, PRR activation may also affect the activity of ion channels on the mitochondrial membrane, alter the mitochondrial membrane potential, and thereby impair mitochondrial function, leading to mtDNA influx from mitochondria into the cytoplasm. However, relevant research is limited, and the specific mechanisms require further investigation.

### Clinical translation of membrane permeability-related mt-DAMPs release

Abnormal mitochondrial membrane permeability plays a critical role in the progression of atherosclerosis by regulating the release of mitochondrial (mt)–DAMPs, and its effects are strikingly threshold dependent. Transient and limited opening of the mPTP leads to low–level release of mt–DAMPs (e.g. short mtDNA fragments, mitochondrial ROS), which activates the cytosolic cGAS–STING pathway and NLRP3 inflammasome, promoting type I INF responses and vascular inflammation [66, 101]. In contrast, persistent mPTP opening results in massive release of cytochrome c, intact mtDNA, and other factors, causing collapse of the mitochondrial membrane potential, ATP depletion, and subsequent apoptosis or necrosis [137–139].

This threshold undergoes dynamic changes across different stages of athersclerosis. In early plaques, oxidative stress and cholesterol loading (e.g. oxLDL) lower the mPTP opening threshold, rendering macrophages more prone to mt–DAMPs release and pro–inflammatory polarization [7]. During plaque instability or acute events such as myocardial infarction, local calcium overload and ROS bursts further reduce the threshold, accelerating cytosolic translocation of mtDNA and inflammatory amplification [16]. Notably, loss of MICU1 or aberrant METTL4–mediated 6mA modification of mtDNA in ECs exacerbates mitochondrial calcium homeostasis imbalance, shifts the activation threshold leftward, and promotes vascular inflammation and plaque progression [16, 140].

For clinical translation, circulating mtDNA levels in serum, platelet mPTP opening sensitivity, CypD acetylation status, and levels of mitochondrial membrane proteins (e.g. VDAC1, Mitochondrially encoded cytochrome c oxidase II (MT–CO2)) in exosomes have been explored as potential biomarkers reflecting mitochondrial membrane permeability [141, 142]. Targeted intervention strategies focus on the following. (i) mPTP inhibitors (e.g. cyclosporine A alleviates mitochondrial swelling and cell death by suppressing CypD activity [143, 144]); blockers of VDAC1 oligomerization to inhibit non–canonical STING–PERK pathway activation [145]. (ii) Modulation of mitochondrial calcium uptake (e.g. targeting MICU1) or enhancement of mitochondrial quality control (e.g. promoting fusion or inhibiting excessive fission) to preserve membrane integrity [16, 52]. (iii) Recent studies indicate that restoring mitochondrial function in macrophages sustains their anti–inflammatory and cholesterol efflux capacities, representing a promising strategy to delay atherosclerosis progression. Meanwhile, precise regulation of mt–DAMPs release nodes (e.g. mPTP, OMM permeabilization), combined with nanocarrier–mediated delivery to improve drug targeting, is emerging as a new focus for mitochondria–targeted therapy in cardiovascular diseases [146, 147]. Future studies are required to further define the threshold windows at distinct pathological stages and promote the clinical implementation of personalized intervention strategies.

## Mitochondrial quality control as a key factor in alleviating innate immune imbalance in atherosclerosis

### Cellular responses to mitochondrial homeostasis imbalance

During the progression of atherosclerosis, disruption of mitochondrial homeostasis triggers multilayered cellular adaptive mechanisms to preserve internal stability. As a core defensive measure, mitophagy plays an indispensable role in curbing excessive innate immune activation caused by leaked mtDNA through selectively eliminating dysfunctional mitochondria [148–150]. Impaired mitophagy leads to aberrant mitochondrial accumulation, which exacerbates oxidative stress and inflammation, and has been directly linked to endothelial dysfunction and enhanced pathological mitochondrial fission in atherosclerosis mouse models [151].

Beyond canonical mitophagy, cells expel mitochondrial components via the secretion of mitochondria-derived vesicles (MDVs). This mechanism, complementary to mitophagy, has been shown to cooperatively maintain mitochondrial homeostasis in skeletal muscle cells and may participate in immune signaling regulation [152]. Notably, recent studies have revealed that mitochondrial nucleoids can be selectively removed through an endosome-dependent mitophagy pathway: upon mtDNA damage, mitochondrial nucleoids are sequestered outside the mitochondrial network and precisely degraded via the endo–autophagic route, providing a novel perspective for understanding mtDNA quality control [153, 154].

The coordinated action of these multi–tiered disposal systems not only prevents mtDNA released from damaged mitochondria from activating innate immune pathways such as cGAS–STING [155, 156], but also establishes a theoretical foundation for developing mitochondria–targeted therapeutic strategies against atherosclerosis [151, 157].

**Figure 3:**
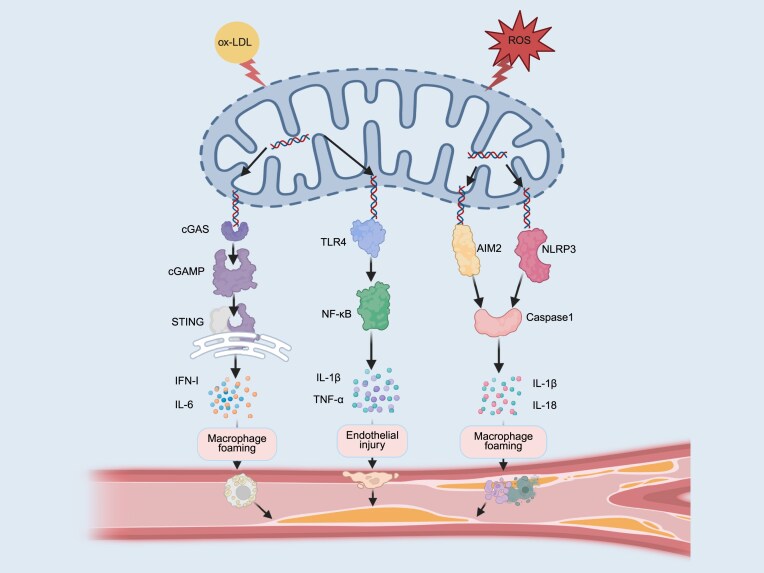
Mitochondrial DNA release activates innate immunity as a key factor in the onset and progression of atherosclerosis. During the initiation and progression of atherosclerosis, mitochondrial damage represents a key event triggering a series of pathological responses, characterized by altered membrane permeability and metabolic disturbances. This damage lead to the release of mt-DAMPs, primarily including mtDNA and mitochondrial ROS (mtROS), which initiate cascading inflammatory responses by activating PRRs, thereby promoting atherosclerosis progression. Specifically, mtDNA can bind to TLR4 to activate NF-κB, inducing the release of IL-1β and tumor necrosis factor-α (TNF-α), which contribute to lipid accumulation and exacerbate endothelial injury. Concurrently, cytoplasmic mtDNA activates cGAS to generate cyclic GMP-AMP (cGAMP); cGAMP then binds to STING, inducing the production of type I interferon (IFN-I) and IL-6, which promote macrophage foam cell formation and plaque instability. mtROS and mtDNA can activate the NLRP3 (NOD-, LRR-, and pyrin domain-containing protein 3) inflammasome, leading to the release of IL-1β and IL-18, and triggering pyroptosis to amplify inflammation and drive atherosclerosis plaque accumulation. Cytoplasmic mtDNA also activates AIM2 to form inflammasomes, which recruit and activate caspase-1; activated caspase-1 cleaves pro-IL-1β to release mature IL-1β, thereby inducing pyroptosis and thinning the plaque fibrous cap. Collectively, the TLR4 pathway, cGAS–STING pathway, NLRP3 inflammasome, and AIM2 inflammasome act through their respective mechanisms to ultimately promote atherosclerosis plaque formation. The image was generated with full licensed BioRender.com.

However, the STING pathway serves not only as a critical innate immune sensor but also as a potent regulator of mitophagy. Emerging evidence demonstrates that STING activation suppresses mitophagy, resulting in the accumulation of damaged mitochondria and subsequent release of mtDNA into the cytoplasm, which further amplifies cGAS–STING signaling and forms a pro–inflammatory positive feedback loop (Fig. 3) [158, 159]. Conversely, under non–pathological conditions, STING promotes mitophagy to maintain mitochondrial homeostasis and restrict excessive type I IFN responses [160].

Furthermore, interventions such as exercise can suppress STING activation by enhancing mitophagy, thereby reducing neuroinflammation and pyroptosis [161]. Therefore, STING exerts a dual regulatory role in mitophagy under distinct physiological or pathological contexts, and its precise modulation is essential for restoring innate immune balance in atherosclerosis.

### Mitophagy eliminates mitochondrial damage caused by innate immune imbalance in atherosclerosis

Mitochondrial damage induced by innate immune imbalance in atherosclerosis constitutes a key pathogenic driver. As a core intracellular mechanism maintaining mitochondrial homeostasis, mitophagy is indispensable for the removal of damaged mitochondria [162]. This process includes the canonical ubiquitin–dependent pathway mediated by PINK1–Parkin, as well as ubiquitin–independent pathways governed by mitophagy receptors such as BNIP3, NIX, and FUNDC1, which collectively preserve mitochondrial quality and cellular homeostasis [163]. Further investigation of its regulatory mechanisms in atherosclerosis will provide novel potential targets for therapeutic intervention.

#### PINK1/Parkin-mediated mitophagy

**Figure 4:**
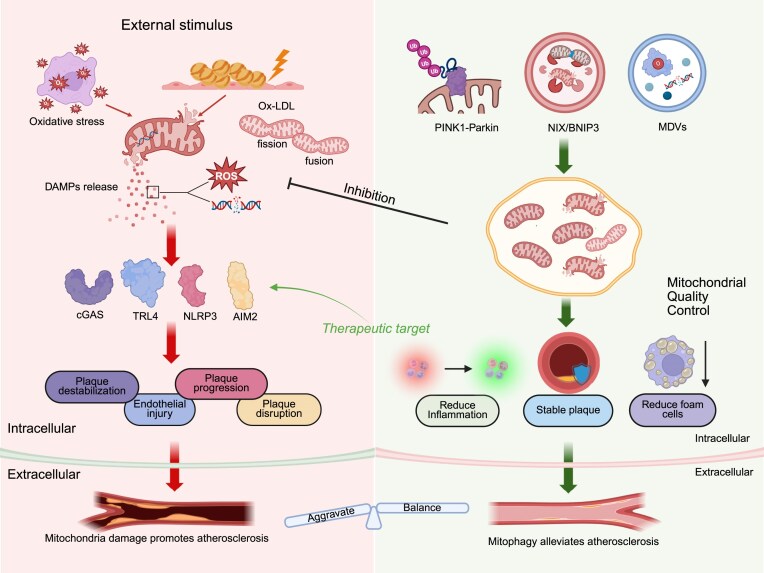
Dynamic regulation of mitochondrial damage and quality control in atherosclerosis. When external stimuli such as oxidative stress and oxLDL induce mitochondrial damage, damaged mitochondria release substances including mtDNA. These molecules can activate innate immunity-related pathways, such as those mediated by TLR4, cGAS, NLRP3, and AIM2, thereby leading to endothelial injury, plaque destabilization, plaque progression, and plaque disruption, which collectively drive the progression of atherosclerosis. In contrast, mitophagy exerts its effects through pathways such as the PINK1-Parkin pathway, autophagic receptors (e.g. NIX, BNIP3), and MDVs to clear damaged mitochondria, reduce the release of pro-inflammatory signals, alleviate inflammation, promote plaque stabilization, and decrease foam cell formation, thereby mitigating the onset and development of atherosclerosis. The image was generated with full licensed BioRender.com.

**Figure 5:**
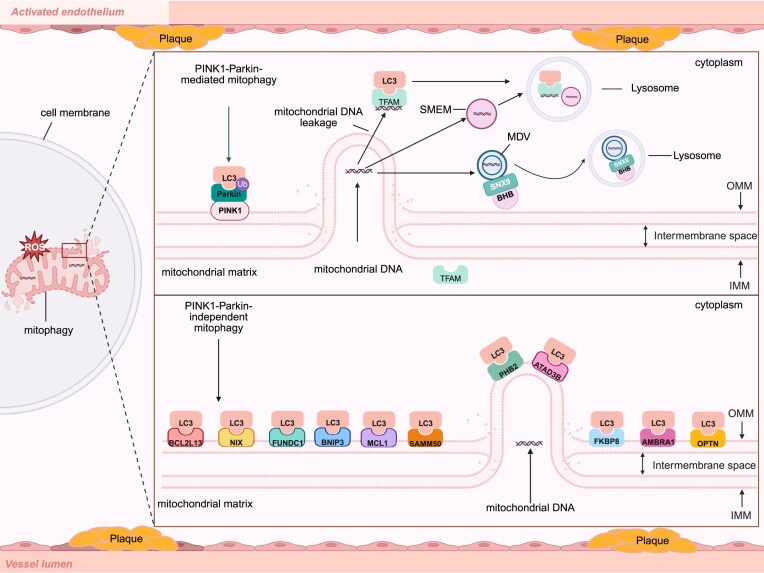
Mitophagy clears damaged mitochondria during the pathological process of atherosclerosis. In cells within atherosclerotic lesion areas (vascular lumen and perivascular plaque regions), damaged mitochondria are cleared through three major pathways to regulate pathological damage in atherosclerosis. (i) Ubiquitination (Ub)-dependent mitophagy mediated by the phosphatase and tensin homolog (PTEN)-induced kinase 1 (PINK1)-Parkin pathway. PINK1 stabilizes on the OMM. Parkin is phosphorylated at the Ser65 site, which activates its E3 ubiquitin ligase activity, catalyzing the Ub of OMM proteins and forming polyubiquitin chain signals characterized by phospho-Ser65-ubiquitin (pS65-Ub). This process further recruits the autophagic protein LC3, enabling damaged mitochondria to be encapsulated by autophagosomes and then fused with lysosomes for degradation. (ii) Mitophagy mediated by autophagy receptors. A variety of autophagy receptors localized to mitochondrial membranes initiate mitophagy by directly binding LC3 via their LC3-interacting regions (LIRs). These receptors include those localized to the OMM (BCL2L13, NIX, FUNDC1, BNIP3, MCL1, SAMM50, FKBP8, AMBRA1, and OPTN) and those localized to the IMM (PHB2 and ATAD3B). (iii) Mitochondrial clearance mediated by MDVs. These MDVs are directionally transported to lysosomes via mediation by the sorting adaptor protein sorting nexin 9 (SNX9). Additionally, the ketone body metabolite β-hydroxybutyrate (BHB) dynamically regulates MDV biogenesis efficiency by modifying SNX9 through lysine β-hydroxybutyrylation (Kbhb). Mitochondrial transcription factor A (TFAM) can leak into the cytoplasm along with mtDNA acting as an autophagy receptor. TFAM binds to the autophagic protein LC3B via its LIR, mediating the clearance of cytoplasmic mtDNA through the lysosomal pathway. The image was generated with full licensed BioRender.com.

Upon mitochondrial damage, the PINK1 kinase is stabilized on the OMM and activates the E3 ubiquitin ligase Parkin through phosphorylation at Ser65. Activated Parkin then catalyzes ubiquitination of OMM proteins (e.g. Mfn1/2, VDAC1), generating polyubiquitin chain signals characterized by phosphorylated ubiquitin (pS65-Ub) [164]. In atherosclerosis, the PINK1-Parkin pathway removes damaged mitochondria by regulating mitophagy, thereby alleviating innate immune imbalance caused by mitochondrial dysfunction. As a mitochondrial damage sensor, PINK1 accumulates on the OMM upon mitochondrial membrane potential depolarization and activates the E3 ubiquitin ligase activity of Parkin by phosphorylating ubiquitin and the ubiquitin-like domain of Parkin [165, 166]. Activated Parkin subsequently ubiquitinates mitochondrial proteins, marking damaged mitochondria for degradation via the LC3-dependent autophagosome–lysosome pathway (Fig. 4) [167, 168]. This process reduces the release of ROS and mtDNA from injured mitochondria; mtDNA can further exacerbate inflammatory responses by activating innate immune pathways such as cGAS–STING [169, 170]. Studies have shown that Parkin deficiency suppresses mitophagy in cardiomyocytes, leading to the accumulation of pro-inflammatory factors (e.g. those related to the NF-κB pathway) and aggravated cardiac dysfunction (Fig. 4) [171]. In addition, the PINK1/Parkin pathway modulates macrophage antigen presentation by regulating mitochondrial quality control (Fig. 5). Its deficiency promotes aberrant crosstalk between macrophages and T cells, exacerbating the immune microenvironment imbalance associated with atherosclerosis [170]. Recent findings indicate that inhibition of USP30, a deubiquitinating enzyme, restores mitophagy and improves mitochondrial respiratory function in PINK1/Parkin-deficient cells [172], whereas calcineurin promotes mitochondrial translocation of Parkin and enhances basal mitophagy through a PINK1-independent mechanism [166]. Collectively, these mechanisms demonstrate that the PINK1-Parkin pathway protects the homeostasis of vascular ECs and immune cells by eliminating dysfunctional mitochondria, reducing pro-inflammatory signaling and apoptosis, and thereby delaying the progression of atherosclerosis [85, 173].

#### Autophagy receptor-dependent mitophagy

In contrast to ubiquitin-dependent mitophagy mediated by PINK1/Parkin, mitochondria harbor numerous proteins containing LC3-interacting regions that act as mitophagy receptors. These receptors can directly bind to LC3 without prior ubiquitination to initiate mitophagy [174]. MDVs participate in the selective degradation of mitochondrial components by carrying such proteins or acting in concert with receptors to bind LC3 in a ubiquitin-independent manner [175].

NIX (also known as BCL2 interacting protein 3 like(BNIP3L)) is a receptor-mediated mitophagy receptor localized to the OMM and plays a critical role in mitochondrial quality control in cardiovascular diseases including atherosclerosis. Mechanistically, NIX binds to ATG8 family proteins (e.g. LC3B, GABA type A receptor-associated protein like 1 (GABARAPL1)) via its LC3-interacting region (LIR) and cooperatively induces mitophagy through its minimal essential region, thereby eliminating damaged mitochondria and suppressing mtROS accumulation and subsequent inflammatory and oxidative stress injury (Fig. 5) [176, 177]. In atherosclerosis models, downregulation of NIX in ECs, VSMCs, and macrophages is closely associated with mitochondrial homeostasis imbalance and increased pathological mitochondrial fission, suggesting a protective role of NIX-mediated mitophagy [151, 178]. Animal studies have shown that Fbxl4-knockout mice exhibit excessive mitophagy due to NIX/BNIP3 accumulation, resulting in perinatal lethality, whereas NIX knockout reverses this phenotype, indicating that NIX levels must be tightly regulated to maintain mitochondrial homeostasis [179, 180]. Furthermore, preclinical studies have demonstrated that natural compounds such as tanshinone I (Tan I) can induce mitophagy by upregulating NIX/BNIP3 and inhibit cervical cancer metastasis [181], suggesting therapeutic potential of targeting the NIX pathway. However, there are currently no clinical trials directly targeting NIX, and most agents remain at the cellular or animal validation stage [182]. As a potential biomarker, NIX expression in platelets is associated with lifespan regulation and may reflect systemic mitophagy status [182]. Notably, animal models cannot fully recapitulate the complex immune microenvironment and long-term disease course of human atherosclerosis, and NIX function may exhibit heterogeneity across different cell types, which limits its clinical translation. Future studies are needed to develop specific NIX modulators and validate their reliability as markers of mitophagy activity in human samples.

FUNDC1 is a receptor protein localized to the OMM and plays a central role in mitophagy. In atherosclerosis, FUNDC1 maintains mitochondrial homeostasis and reduces innate immune imbalance by mediating the clearance of damaged mitochondria via mitophagy. Studies have shown that FUNDC1 acts through multiple mechanisms. (i) In cardiac microvascular ECs, age-dependent reduction in FUNDC1 levels leads to decreased mitophagy, triggering endothelial senescence and mitochondrial dysfunction, thereby promoting atherosclerosis-related pathological changes [63, 183]. (ii) FUNDC1 promotes autophagosome formation (e.g. assembly of the ATG5-ATG12/ATG16L1 complex) by restoring mitochondria–ER contact sites structure, thereby enhancing macroautophagy to eliminate damaged mitochondria [184]. (iii) Under oxidative stress, FUNDC1 directly mediates mitophagy by recruiting LC3B to mitochondria (dependent on its dephosphorylation) (Fig. 5), reducing mtROS accumulation and lipid peroxidation, thereby inhibiting endothelial ferroptosis and microcirculatory dysfunction [185, 186]. Furthermore, loss or functional inhibition of FUNDC1 exacerbates mitochondrial quality control imbalance, leading to the accumulation of damaged mitochondria in inflammatory macrophages, promoting pro-inflammatory cytokine release and atherosclerotic plaque instability [187]. Studies in animal models have shown that FUNDC1-knockout mice are more susceptible to impaired mitochondrial clearance, ROS accumulation, and vascular remodeling under HFD or hypoxic conditions, indicating its critical regulatory role in atherosclerosis progression [188, 189]. Preclinical studies have demonstrated that targeted activation of FUNDC1 (e.g. using the mitophagy activator urolithin A or adeno-associated virus (AAV)-mediated FUNDC1 overexpression) significantly improves endothelial function and alleviates vascular remodeling [183, 186], suggesting FUNDC1 as a potential therapeutic target for atherosclerosis.

Multiple additional mitophagy receptors also coordinately regulate mitochondrial quality control through distinct pathways and participate in the pathogenesis of atherosclerosis. BNIP3, a hypoxia-inducible OMM protein, directly interacts with LC3/GABARAP family proteins under stress to mediate ubiquitin-independent mitochondrial clearance. Its downregulation is closely associated with macrophage polarization toward a pro-inflammatory M1 phenotype, thereby promoting plaque instability [190, 191]. PHB2, located in the IMM, becomes exposed to the outer membrane upon mitochondrial membrane potential loss and initiates Parkin-dependent mitophagy by binding to LC3, which is essential for maintaining EC and VSMC homeostasis. BCL2L13, a functional mammalian homolog of yeast Atg32, can also directly recruit autophagic machinery to damaged mitochondria and participate in ubiquitin-independent pathways [192, 193]. In addition, MCL1, ATAD3B, and SAMM50 are primarily involved in maintaining mitochondrial structure and dynamics [150, 194, 195], but recent studies suggest that they may also indirectly affect mitophagic efficiency. For example, as a component of the mitochondrial fission complex, SAMM50 dysfunction leads to pathological mitochondrial fragmentation, which interferes with normal mitophagy and exacerbates endothelial injury [151]. In summary, these receptors coordinately regulate mitochondrial quality control through ubiquitin-dependent or -independent pathways, forming a critical defense line against excessive innate immune activation in atherosclerosis (Fig. 5).

#### Role of MDVs in the clearance of damaged mitochondria in atherosclerosis

Mitochondrial-derived vesicles exert a critical mitochondrial quality control function in atherosclerosis by removing damaged mitochondrial components caused by innate immune activation. Under mild mitochondrial stress, MDVs selectively encapsulate damaged mtDNA, proteins, and lipids and deliver them to the lysosomal or endosomal system for degradation, thereby preserving mitochondrial homeostasis and suppressing inflammatory responses (Fig. 5) [196, 197]. Notably, mtDNA, as an mtDAMP, can activate the NLRP3 inflammasome and promote IL-1β secretion. Marked mtDNA damage and mitochondrial respiratory dysfunction have been well documented in atherosclerotic plaques [66, 198, 199]. Preclinical studies in mouse models of atherosclerosis have demonstrated that reducing mtDNA damage improves mitochondrial respiratory function, reduces necrotic core size, and thickens the fibrous cap, indicating therapeutic potential of targeting mitochondrial repair [198]. In addition, circulating cell-free DNA (cfDNA)—including damaged products of both mitochondrial and nuclear genomic origin—has been explored as a potential biomarker for atherosclerosis [200]. However, translational limitations exist from animal models to humans. For instance, mice lack lipoprotein metabolism and chronic inflammatory microenvironments similar to those in humans, which may compromise the extrapolation of MDV-mediated protective mechanisms [201].

### Clearance of mitochondrial nucleoids in atherosclerosis

Mitochondrial nucleoids are specialized structures within the mitochondrial matrix that are primarily responsible for the packaging, protection, and transcriptional regulation of mtDNA [202]. Nucleoid-mediated targeted mtDNA degradation is a dedicated mechanism of mitochondrial quality control, primarily removing damaged mtDNA through specific membrane remodeling and endosomal pathways to prevent its activation of innate immunity [203]. mtDNA damage induces substructural changes in nucleoids, and through membrane remodeling and cristae architecture adjustment, promotes nucleoids to approach the OMM. This process is coordinately controlled by ATAD3 and SAMM50 proteins: ATAD3 maintains cristae architecture and nucleoid interactions, while SAMM50 acts as a gatekeeper to influence BAK protein aggregation and regulate nucleoid release into the cytoplasm [204]. Upon damage, SAMM50 dissociates from the axis, leading to the recruitment of nucleoids to endosomal compartments and the initiation of degradation via endosomal trafficking. This mechanism relies on the interaction between SAMM50 and the retromer protein VPS35. VPS35 mediates endosomal maturation, transforming early endosomes into late autophagy vesicles, and guiding mtDNA-containing nucleoids to migrate to lysosomes for targeted degradation [153]. Nucleoid degradation mainly occurs through the endosomal-mitophagy pathway, independent of classical macroautophagy but requiring lysosomal function and the involvement of the key protein ATG5. When mtDNA undergoes replication errors or stress, nucleoids are packaged into endosomal vesicles and cleared through an “adaptive mitochondrial–endosomal quality control pathway”. This process is termed nucleoid-phagy: mitochondrial transcription factor A translocates to the cytoplasm along with mtDNA and is mislocalized under stress conditions; the nucleoid-phagy mechanism removes these mtDNA fragments in an endosome-dependent manner to maintain cellular homeostasis (Fig. 5) [205]. Under specific circumstances, replication-incompetent enlarged nucleoids are continuously recruited to early endosomes and then converted into late endosomes, with mtDNA fragments released through endosomal rupture—a risk avoided through targeted degradation: the endosomal pathway directs nucleoids to autophagy vesicles for degradation by lysosomal enzymes [206]. This mechanism removes mutated mtDNA without affecting the copy number of normal mtDNA; for example, stimulating lysosomal activity can selectively clear defective mtDNA and improve mitochondrial function [207]. The nucleoid-mediated degradation mechanism can restore immune balance: clearing mislocalized mtDNA through “nucleoid-phagy” reduces cytoplasmic mtDNA accumulation, maintains cellular homeostasis, and prevents cell death or inflammatory spread [208].

In addition to autophagic clearance pathways, nuclease-mediated pathways also represent an essential mechanism for mtDNA degradation. When mtDNA sustains damage such as double-strand breaks that cannot be efficiently repaired or removed, mitochondrial dysfunction is triggered and mtDNA is released into the cytosol, thereby exacerbating chronic inflammatory responses in atherosclerosis [66]. To prevent the accumulation of aberrant mtDNA, cells rely on specific nucleases for the rapid degradation of damaged mtDNA. Studies have demonstrated that core enzymes involved in mtDNA replication, including POLG, TWNK, and MGME1, also participate in the degradation of damaged mtDNA [209]. Furthermore, multiple mitochondrial nucleases, such as EXOG, APEX2, ENDOG, FEN1, and DNA2, have been shown to regulate mtDNA metabolism, yet their specific roles in the context of atherosclerosis remain to be fully elucidated [210].

### Mitochondrial transfer in atherosclerosis

Cells reduce the accumulation of intracellular DAMPs by extruding damaged mitochondria (e.g. via exosomes or direct release). For instance, macrophages in atherosclerosis plaques can eliminate dysfunctional mitochondria through mitochondrial extrusion, avoiding the persistent activation of the NLRP3 inflammasome [12]. Experimental evidence has shown that the use of mitochondria-targeted hydrogen sulfide (H₂S) donors (e.g. AP39) can reduce mitochondrial damage within plaques and significantly alleviate atherosclerosis [211].

In the atherosclerosis microenvironment, healthy mitochondria can be transferred from mesenchymal stem cells to damaged ECs via tunneling nanotubes (TNTs) or exosomes, restoring their bioenergetic and barrier functions [212, 213]. This transfer relies on gap junction proteins (e.g. Cx43) and actin cytoskeleton reorganization [214].

Macrophages that receive healthy mitochondria can reverse “pro-inflammatory polarization” (e.g. the conversion from the M1 to the M2 phenotype). For example, transferred mitochondria inhibit glycolysis by enhancing OXPHOS, reduce IL-1β secretion, and simultaneously promote the production of anti-inflammatory factors (e.g. IL-10) [17]. In obesity-associated atherosclerosis models, mitochondrial transfer has also been shown to ameliorate adipose tissue inflammation [215].

Mitochondrial transplantation has demonstrated efficacy in mouse atherosclerosis models. Direct delivery of healthy mitochondria to plaques reduces ROS production, restores macrophage phagocytic capacity, and promotes plaque stability [216]. For instance, the use of mitochondria derived from mesenchymal stem cells can significantly reduce aortic plaque area [215].

## Specific mitochondrial dysfunction at distinct stages of atherosclerosis

**Figure 6:**
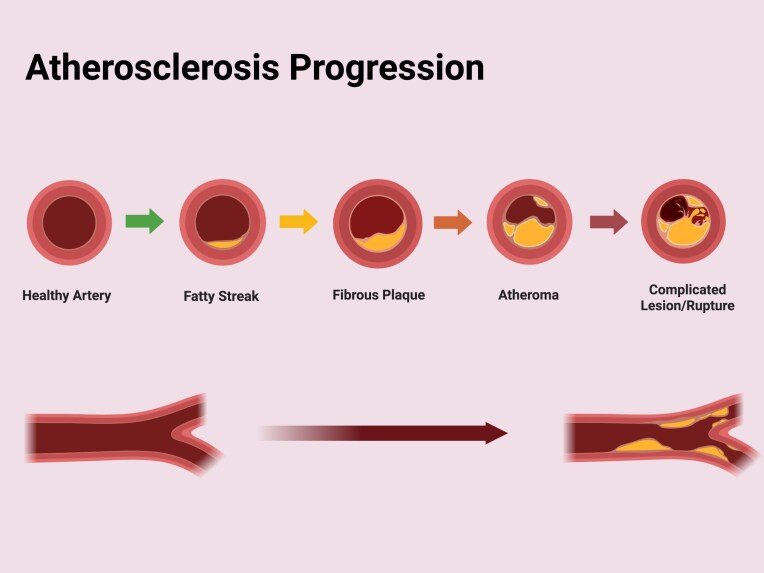
Dynamic progression of atherosclerosis. This figure illustrates the typical pathological progression of atherosclerosis. The figure sequentially depicts the five major stages of lesion development from a healthy artery, including fatty streak, fibrous plaque, atheroma, and ultimately the formation of a complicated lesion or lesion rupture. The progression is presented through serial schematic cross-sections of arteries, where yellow regions represent lipid deposition and dark red regions indicate the necrotic core or plaque contents. The image was generated with full licensed BioRender.com.

Atherosclerosis progression can be broadly divided into three stages: the early stage, dominated by endothelial mitochondrial dysfunction, which initiates the formation of fatty streaks; the progressive stage, driven by macrophage mitochondrial dysfunction that promotes inflammation and lipid accumulation, leading to fibrous plaques and atheromas; and the advanced plaque and complication stage, culminating in complicated lesions or rupture (Fig. 6), during which mitochondrial abnormalities in T cells and VSMCs contribute to immune imbalance and plaque rupture. The dominant cell types at distinct stages exhibit characteristic patterns of mitochondrial dysfunction.

### Mitochondrial dysfunction in the early stage of atherosclerosis

At the initiation stage of atherosclerosis, vascular endothelial injury represents a key event. Mitochondrial dysfunction in ECs is mainly characterized by disrupted mitochondrial calcium homeostasis, excessive ROS production, and abnormal mitochondrial dynamics. Studies have revealed that endothelial-specific MICU1 maintains mitochondrial Ca²⁺ homeostasis, thereby suppressing vascular inflammation and atherosclerosis progression. Endothelial-specific MICU1 knockout mouse models exhibit disrupted endothelial mitochondrial function, elevated ROS levels, and increased plaque formation, indicating a protective role of MICU1 [16]. This mechanism was validated using genetically modified mouse models.

### Macrophage mitochondrial dysfunction drives inflammation and lipid accumulation during atherosclerosis progression

With the progression of atherosclerotic lesions, monocytes infiltrate and differentiate into macrophages, which internalize oxLDL to form foam cells. At this stage, mitochondrial dysfunction in macrophages becomes particularly prominent, typically manifesting as impaired mitochondrial function, damaged respiratory chain, decreased NAD⁺ levels, metabolic shift toward glycolysis, and a pro-inflammatory phenotype, all of which collectively promote plaque progression and vulnerability [7]. For example, impaired translation of mitochondrial alanyl-tRNA synthetase 2 (Aars2) leads to defective mitochondrial protein synthesis, triggers NAD⁺ depletion, activates the integrated stress response, and ultimately compromises macrophage phagocytic function, thereby promoting plaque instability [217]. This mechanism was verified in mouse bone marrow-derived macrophages and HFD-induced atherosclerosis mouse models, representing combined cellular and animal experiments. NADPH oxidase 4 is upregulated in aging-related atherosclerosis and exacerbates the pro-inflammatory phenotype of macrophages by inducing mitochondrial ROS production. Inhibition of NADPH oxidase 4 improves mitochondrial function and alleviates vascular inflammation, as confirmed in ApoE⁻/⁻ mice, constituting an interventional animal study [218]. Furthermore, abnormal metabolic stress (e.g. lipid overload) can directly induce macrophage mitochondrial injury, thereby impairing phagocytosis and inflammatory regulation [14]. Notably, iron deficiency has been shown to mediate mitochondrial and phagocytic dysfunction in macrophages induced by specific quinone compounds, whereas iron supplementation partially reverses these effects [219]. Of note, mitochondrial translation efficiency is critical for macrophage function. Sufficient mitochondrial translation promotes the formation of mature phagocytic macrophages and exerts anti-atherogenic effects [217].

### Mitochondrial abnormalities in T cells and smooth muscle cells as key determinants in advanced plaque and complication stages

In advanced atherosclerosis, unstable function of regulatory T cells is closely associated with activation of the mitochondrial–endoplasmic reticulum stress axis. Regulatory T cell numbers are reduced in plaques from patients with coronary artery disease, and combined mitochondrial dysfunction and endoplasmic reticulum stress result in the loss of immunosuppressive function [220]. In addition, overactivation of the RNA-binding protein LARP4 in exhausted CD8⁺ T cells enhances the translation of nuclear-encoded OXPHOS mRNAs, disrupts the stoichiometric balance of mitochondrial complex subunits, and induces mitochondrial dysfunction [221]. This mechanism has been established in tumor models, but its role in atherosclerosis remains to be validated and is currently supported only by cellular experimental inference. VSMCs play a critical role in fibrous cap formation. Mitochondrial dysfunction in VSMCs leads to phenotypic switching, reduced migration, increased apoptosis, and weakened plaque stability. Although mechanistic studies remain limited, existing evidence indicates that imbalanced mitochondrial dynamics (e.g. hyperactivated Drp1) contributes to VSMC dysfunction [201].

## Concluding remarks

**Figure 7:**
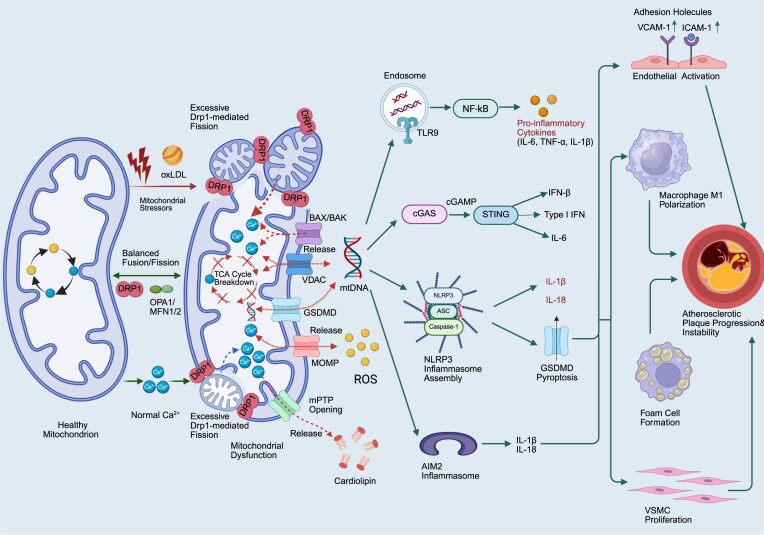
Dysregulation of mitochondrial homeostasis regulates the progression of atherosclerosis. This figure illustrates how mitochondrial metabolic imbalance, mitochondrial dynamics dysregulation, and calcium (Ca²⁺) homeostasis disruption induce mitochondrial content release, innate immune receptor activation, and pro-inflammatory responses through multiple molecular pathways, thereby regulating the progression of atherosclerosis. Left section—healthy mitochondria and physiological homeostasis—depicts the dynamic balance of healthy mitochondria (balanced fusion/fission), maintained by mitochondrial fusion proteins 1 (OPA1) and MFN1/2, while mitochondrial fission is regulated by DRP1. Intracellular Ca²⁺ is maintained at normal levels (normal Ca²⁺), and the TCA cycle and OXPHOS operate normally, sustaining energy and metabolic homeostasis. Mid-left section—inducing factors and mitochondrial damage. oxLDL and other mitochondrial stressors cause excessive activation of DRP1, leading to excessive DRP1-mediated fission and mitochondrial fragmentation. Concurrently, Ca²⁺ homeostasis disruption is characterized by abnormal Ca²⁺ influx/accumulation between mitochondria and the cytoplasm, resulting in decreased mitochondrial membrane potential, impairment of key TCA cycle enzymes, metabolic imbalance (TCA cycle breakdown), and increased ROS production. Metabolic imbalance is also accompanied by the accumulation of metabolites (e.g. succinate) and insufficient adenosine triphosphate (ATP) generation. Alteration of mitochondrial membrane permeability and content release occurs when OMM proteins Bcl-2-associated X protein (BAX)/Bcl-2 homologous antagonist/killer (BAK) mediate MOMP; the opening of VDAC1 and mPTP facilitates the release of mtDNA, cardiolipin, cytochrome c, and other mitochondrial contents into the cytoplasm or endosomes. GSDMD forms membrane pores that mediate cytoplasmic content release and trigger programmed inflammatory cell death (pyroptosis). These events are accompanied by elevated ROS levels, amplifying intracellular inflammatory signals. Mid-right section—innate immune pathways activated by mtDNA and other mitochondrial damage-associated molecules: cGAS recognizes cytoplasmic mtDNA, synthesizes cyclic GMP-AMP (cGAMP), and activates STING, which in turn recruits and activates TBK1 and interferon regulatory factor 3 (IRF3), inducing type I IFNs, IFN-β, and pro-inflammatory cytokines such as IL-6. TLR9 recognizes mtDNA fragments in endosomes, activates NF-κB, and promotes the expression and release of pro-inflammatory cytokines including IL-6, tumor necrosis factor-α (TNF-α), and IL-1β. The cytoplasmic NLRP3 inflammasome assembles, recruits apoptosis-associated speck-like protein containing a CARD (ASC) and caspase-1, catalyzes the maturation of pro-IL-1β and pro-IL-18 to promote their secretion, and mediates pyroptosis/pyroptosis-like release via GSDMD. The AIM2 inflammasome can also be activated by mtDNA, facilitating the release of IL-1β and IL-18. Right section—downstream tissue/cellular effects and plaque evolution. The aforementioned mtDNA-related cGAS–STING pathway, TLR9, NLRP3/AIM2 inflammasomes, and the production of associated pro-inflammatory cytokines induce systemic and local inflammatory responses: vascular ECs are activated and upregulate adhesion molecules, including VCAM-1 and intercellular adhesion molecule-1 (ICAM-1), promoting monocyte/leukocyte adhesion and infiltration; the inflammatory microenvironment drives macrophage polarization toward the pro-inflammatory M1 phenotype and accelerates foam cell formation; VSMC proliferation is also induced; collectively, these changes drive atherosclerotic plaque growth, lipid core accumulation, and structural instability (plaque progression and instability). This figure presents a sequential cascade of events: starting from mitochondrial dynamics and metabolic/Ca²⁺ dysregulation, progressing through the disruption of mitochondrial membrane integrity and release of contents (mtDNA, cardiolipin, ROS, etc.), activating multiple innate immune sensors (cGAS–STING, TLR9, NLRP3 inflammasome, AIM2), and ultimately leading to endothelial activation, inflammatory macrophage polarization, foam cell formation, and VSMC proliferation through changes in cytokines and cellular behaviors (e.g. IL-1β, IL-18, IL-6, TNF-α, Type I IFN), synergistically promoting the development and destabilization of atherosclerosis. The image was generated with full licensed BioRender.com.

In summary, mitochondrial damage serves as a pivotal inducer of innate immune imbalance, encompassing the activation of TLRs, aberrant signaling of the cGAS–STING pathway, and the activation of NLRP3 and AIM2 inflammasomes in atherosclerosis (Fig. 7) [11, 213, 222]. Mitophagy, through mechanisms including the Pink1-Parkin pathway, autophagy receptor mediation, and MDVs, clears damaged mitochondria, which can effectively alleviate innate immune disorders and thus exert a protective effect against atherosclerosis [178, 223]. Among these, the mechanisms underlying the clearance of mitochondrial contents provide new perspectives for atherosclerosis research. GPR50, as a novel mitophagy receptor, binds to LC3 via its LIR motif, targets damaged mitochondria under stress, and promotes their degradation [224]. Its functional deficiency leads to mitochondrial dysfunction and neuronal developmental disorders, suggesting that similar mechanisms may be involved in the repair process after vascular endothelial cell damage [224]. The selective clearance of IMM fragments mediated by Vesicles Derived from the Inner Mitochondrial Membrane (VDIMs), a microautophagy process dependent on VDAC1 pores and Endosomal sorting complexes required for transport (ESCRT) machinery, may be an important way for arterial wall cells to cope with oxidative stress [225]. TFAM mediates nucleophagy through its LIR motif to clear cytoplasmic mtDNA, limiting the activation of the cGAS–STING pathway, which provides a new target for the regulation of inflammatory responses in atherosclerosis [208, 226, 227]. Additionally, mitochondrial fission process 1(MTFP1) isolates damaged inner membrane substructures into small MTFP1-enriched mitochondria by inhibiting IMM fusion, and promotes their autophagic degradation through LIR binding to LC3, a mechanism crucial for maintaining mtDNA homeostasis [228]. Future exploration of how targeted clearance mechanisms of mitochondrial contents synergistically maintain mitochondrial homeostasis in vascular cells and their roles in the initiation and progression of atherosclerosis plaques may provide new strategies for disease prevention and treatment.

Novel mitochondria may also play a significant role in the progression of atherosclerosis. Novel mitochondria can be defined as functionally specialized subpopulations formed through fusion–fission dynamics, with their core feature lying in the specialized separation of metabolic functions [229]. One subset is mitochondria rich in cristae structures and ATP synthase, primarily responsible for OXPHOS to efficiently generate ATP and meet cellular energy demands [229]. The other subset, which lacks cristae and ATP synthase but retains ETC activity, is enriched in the rate-limiting enzyme for proline/ornithine synthesis, P5CS, and can provide reducing equivalents for reductive biosynthesis by maintaining a relatively high membrane potential [229]. This functional differentiation is not static but achieves dynamic balance through cycles of fusion mediated by mitochondrial fusion proteins and fission driven by Drp1 [230, 231]. This ensures the precise segregation of the two subpopulations within cells, thereby coordinating the demands for energy production and biosynthesis when OXPHOS dependence is enhanced [229]. Additionally, the high membrane potential characteristic of the P5CS-enriched mitochondrial subpopulation provides a potential target for exploring the association between mitochondrial stress and cellular phenotypic switching in atherosclerosis [229]. Implementing mitochondrial quality control tailored to different types of mitochondria is expected to offer new insights into the research on atherosclerosis pathogenesis and the development of intervention strategies.

Similarly, mitophagy as a therapeutic target for atherosclerosis faces multiple challenges. Firstly, there are technical difficulties in the selective clearance of damaged mitochondria. Although known mitophagy receptors (such as BCL2L13 and MCL1) can mediate mitochondrial clearance, their specific regulatory mechanisms have not been fully elucidated, making it difficult to accurately distinguish between damaged and healthy mitochondria [232–234]. Secondly, the microenvironment of atherosclerosis lesions is complex, and oxidative stress and inflammatory responses can interfere with the function of classical pathways such as PINK1/Parkin, reducing mitophagy efficiency. Furthermore, existing drugs (such as small-molecule compounds like UMI-77 and PR-364) can activate mitophagy but may pose a risk of excessive mitochondrial clearance, which in turn exacerbates cellular energy metabolism disorders. Targeted delivery technology is a potential breakthrough. Nanocarriers (such as ROS-responsive liposomes and platelet membrane-coated nanoparticles) can enhance drug accumulation in plaques and reduce non-specific damage through the synergistic effect of regulating mitophagy and antioxidant activity (e.g. superoxide dismutase (SOD)/catalase co-delivery systems) [235–237]. However, achieving spatiotemporally specific activation of mitophagy remains to be resolved. For example, simultaneous blockade of autophagic flux (e.g. using hydroxychloroquine) may lead to the accumulation of mitochondrial fragments and exacerbate oxidative stress [238]. Future efforts are needed to develop dynamic monitoring technologies (such as second near-infrared window (NIR-II) imaging) combined with precise regulatory approaches to balance mitochondrial quality control and cellular homeostasis. In conclusion, overcoming the challenges of selectively clearing damaged mitochondria requires breakthroughs in three aspects: optimizing receptor targeting, improving adaptability to the lesion microenvironment, and developing intelligent drug delivery systems.

The contribution of mitochondrial dysfunction to atherosclerosis is not limited to pathological processes such as pro-inflammatory signaling, oxidative stress, and endothelial injury; critically, it also triggers a series of adaptive and compensatory responses in the organism. First, as a core quality-control mechanism, mitophagy selectively eliminates damaged mitochondria and prevents the release of harmful molecules such as mtDNA, which would otherwise activate innate immune pathways including the cGAS–STING pathway [239]. Meanwhile, in response to mitochondrial stress, cells can enhance protein quality control by activating the mitochondrial unfolded protein response [240] or maintain energy and redox homeostasis via the integrated stress response pathway [241]. In ECs, telomerase reverse transcriptase (TERT) overexpression reduces mitochondrial ROS production and preserves ATP levels, indicating a non-canonical mitochondrial protective function of telomerase [242]. Furthermore, metabolic reprogramming—such as enhanced glycolysis or upregulated pyruvate dehydrogenase activity—also serves as a compensatory mechanism for respiratory chain defects [243]. Notably, mild mitochondrial stress may even elicit beneficial effects, such as increasing mitochondrial mass to sustain functional output [244] or activating the *de novo* serine synthesis pathway to support respiration and lipid homeostasis [245]. These compensatory mechanisms collectively form an intrinsic defense line against mitochondrial damage. However, once injury exceeds the compensatory threshold, the resulting imbalance drives NLRP3 inflammasome activation [6], macrophage polarization, and plaque instability [218]. Thus, atherosclerosis progression is not determined by mitochondrial damage alone, but by the dynamic balance between damage and compensation. Therefore, future therapeutic strategies should not only target the suppression of injury but also focus on enhancing the intrinsic mitochondrial repair and regulatory capacity, to achieve coordinated control of inflammation and tissue protection, thereby restoring this critical balance [246].
